# Tunable Acoustic Metasurface with High-Q Spectrum Splitting

**DOI:** 10.3390/ma11101976

**Published:** 2018-10-14

**Authors:** Shilong Zhai, Kun Song, Changlin Ding, Yuanbo Wang, Yibao Dong, Xiaopeng Zhao

**Affiliations:** Smart Materials Laboratory, Department of Applied Physics, Northwestern Polytechnical University (NWPU), Xi’an 710129, China; dingchanglin@nwpu.edu.cn (C.D.); yuanbowang@mail.nwpu.edu.cn (Y.W.); ybdong@mail.nwpu.edu.cn (Y.D.); xpzhao@nwpu.edu.cn (X.Z.)

**Keywords:** metasurface, tunability, high Q-factor, spectrum splitting

## Abstract

We propose a tunable acoustic metasurface using a nested structure as the microunit, which is constituted by two distinct resonators. Thanks to the coupling resonance for the microunit and by simply adjusting the rotation angle of the inner split cavity, this nested structure provides nearly 2π phase shift. The full-wave simulations demonstrate that the constructed metasurface can be tuned to reflect incident sound waves to different directions in the operation frequency region with a very narrow bandwidth, which is a key functionality for many applications such as filtering and imaging. Meanwhile, the reflected sound waves out of the operation frequency region always remain unchanged. As a result, a high Q-factor spectrum splitting can be realised. The presented metasurface is of importance to develop many metamaterial-based devices, such as tunable acoustic cloaks and acoustic switching devices.

## 1. Introduction

It is always the main theme in the field of acoustics to control and mould the propagation of sound waves. However, the conventional devices are normally bulky and exhibit poor performance at the subwavelength scale due to the diffraction limit. Recent years have witnessed the continuous and robust development of acoustic metamaterials, which are defined as man-made structures of flexible and even novel acoustic effective properties [1,2]. By using subwavelength structures as units, acoustic metamaterials can realise many extraordinary phenomena such as negative refraction, inverse Doppler effect, cloaking, slab focusing, deep-subwavelength imaging, and super absorption [3,4,5,6,7,8,9,10,11,12]. More recently, acoustic metasurfaces, metamaterials of reduced dimension, have attracted much research efforts owing to their ultra-thin thickness. Such metasurfaces are constructed with space variant acoustic resonators with a subwavelength size, whose acoustic response is designed to exhibit desired phase shift by adjusting their geometry. Until now, many works about acoustic metasurfaces have been devoted to investigating new possibilities to manipulate sound waves, such as abnormal reflection [13,14,15], abnormal transmission [16,17,18,19], ultra-thin slab focusing [20], non-diffracting Airy beam [21,22], and ultra-thin cloaking [23]. 

Spectrum splitting has been widely used in the fields of optics and microwave such as spectral component analysis, filtering, optical switching, communication, signal processing, hyperspectral imaging, color holography, and biomedical sensing [24,25,26,27,28]. In conventional optical devices, spectrum splitting is enabled by either geometrical optics or diffractive optics. However, these devices provide limited control in splitting the spectrum significantly due to the dispersive nature of materials. In contrast, optical metasurfaces have the potential to overcome this challenge, because they are able to shape desired wave-fronts and precisely control the phase modulation. 

Quite recently, the spectrum splitting of electromagnetic waves of metasurfaces for both optical and THz regimes have been proposed [29,30,31]. As an analogue of the optical metasurface in the acoustic band, the acoustic metasurface shares similar advantages compared with conventional acoustic devices. However, to the best of our knowledge, the spectrum splitting of acoustic waves with acoustic metasurfaces has rarely been investigated. Besides, the reported acoustic metasurfaces are realised mainly by using space-coiling microunits with relatively complex internal structures, which result in high requirements for machining accuracy. Moreover, the response frequencies of most metasurfaces are fixed and cannot be further adjusted, which largely limits the applications of metasurfaces. Therefore, the need for tuneable metasurfaces is urgent [32,33,34]. Broadband phase modulation by the metasurface with different geometries has been demonstrated to be very useful [35], however, for many applications such as filtering and imaging, narrowband frequency selectivity with a high-Q factor is a key functionality [29].

In this article, we propose and demonstrate a tuneable metasurface within audible frequency regime. The microunit for our metasurface is a simple nested structure consisting of a split cavity and an annular split cavity, which is quite easy about fabrication with high geometric accuracy. The reflection direction of sound waves can be tuned arbitrarily by mechanically adjusting the rotation angles of inner split cavities in the microunits as required. Owing to the strong coupling resonance for the nested structure, the operation bandwidth of this metasurface is quite narrow, and a high-Q (~10^2^) spectrum splitting is realised. Such metasurface designs could easily find applications for wavelength-selective filters, directional emitters, and focusing lenses.

## 2.Results and Discussion

### 2.1. Model Analysis

**Figure 1:**
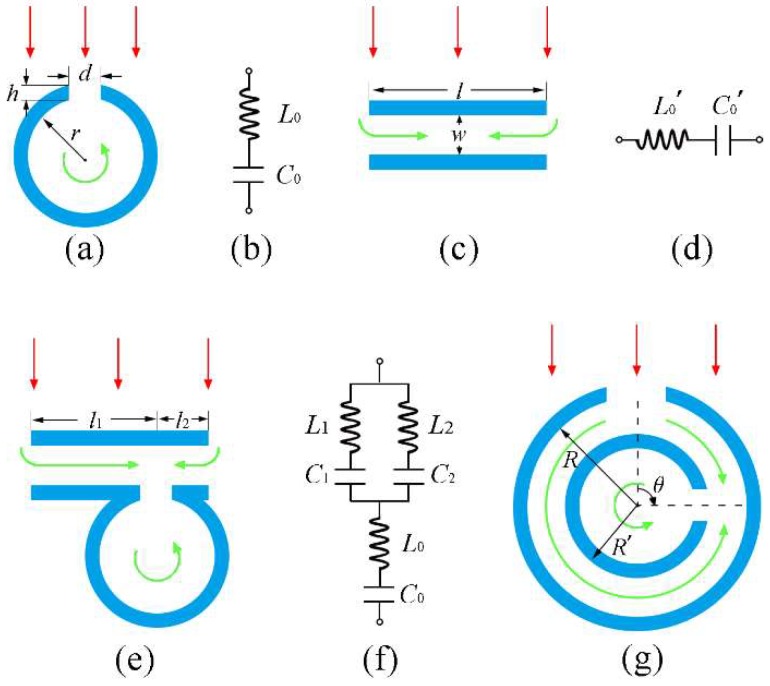
(**a**) and (**b**) Schematic sketch and equivalent circuit of the acoustic split hollow sphere (SHS). (**c**) and (**d**) Schematic sketch and equivalent circuit of the acoustic hollow tube (HT). (**e**) and (**f**) Schematic sketch and equivalent circuit of the coupled structure by SHS and HT. (**g**) Schematic sketch of the optimised model based on (**e**), the inner ring can be spun freely to arbitrary angle *θ* around its centre axis. All the blue areas indicate the acoustic rigid material, and the white areas are filled with air medium. The red arrows denote the incident waves. The green arrows indicate the schematic path of sound waves through the structures.

The key to design the structural unit of a metasurface is to realise a phase shift covering 2π (i.e., 360°) range [19]. For a locally resonant structure, the phase delay of the reflected sound wave generally reaches the maximum near its resonant frequency [36]. Therefore, the emphasis of designing the microunit of metasurface is to find the relationship between its resonant frequency and structural parameters. During our previous studies, we have demonstrated two kinds of acoustic metamaterials based on two acoustic locally resonant meta-atom models (i.e., the split hollow sphere (SHS) [8] and the hollow tube (HT) [11]), respectively. On the basis of the equivalent circuit theory, the cavity and neck of SHS (i.e., a simplified 2D model as shown in Figure 1a) can be regarded as a capacitor with capacitance *C*_0_ and an inductor with inductance *L*_0_, respectively [6,8].

(1) $$C_{0}\propto \frac{V}{\rho_{0}c_{0}^{2}},L_{0}\propto \frac{\rho_{0}h^{\prime }}{S},$$

where $\rho_{0}$ and $c_{0}$ represent the density of air and velocity of sound, respectively. *V* is the volume of the interior cavity. $h^{\prime }$ and *S* are the effective length and the cross-sectional area of the neck, separately. The equivalent circuit of SHS is shown in Figure 1b. Then, the resonant frequency *f*_0_ of SHS can be deduced as follows:

(2) $$f_{0}=\frac{1}{2\pi \sqrt{L_{0}C_{0}}}.$$

Equations (1) and (2) indicate that the acoustic property of SHS greatly depends on its geometric parameters. When the frequency of incident wave approaches the resonant frequency, the sound energy will accumulate in the cavity of SHS. Thus, the vibration direction of nearby air medium will be opposite to that of incident sound wave, which causes obvious phase delay [37]. The 2D model and equivalent circuit of HT are shown in Figure 1c,d, respectively [11]. Similarly, the cavity of HT acts as the series connection of a capacitor with capacitance ${C^{\prime }}_{0}$ and an inductor with inductance ${L^{\prime }}_{0}$.

(3) $$L_{0}{}^{\prime }\propto \frac{\rho_{0}l}{w},C_{0}{}^{\prime }\propto \frac{wl}{\rho_{0}c_{0}^{2}},$$

where *l* and *w* are the length and width of the HT’s cavity, respectively. Based on Equation (3), the resonant frequency ${f^{\prime }}_{0}$ of HT also depends on its geometric parameters. When the frequency of incident wave approaches its resonant frequency, this structure will cause an obvious phase shift as well.

Here, in order to achieve the purpose of narrow bandwidth, we need to build a structural model with a stronger locally resonant effect than both SHS and HT. So, we coupled the SHS with the HT into an integrated structure as illustrated in Figure 1e. This coupled structure can be regarded as two HTs with the lengths of *l*_1_ and *l*_2_ in parallel, then in series with one SHS. The equivalent circuit is shown in Figure 1f. In this case, the inductance *L* and capacitance *C* of this coupled structure can be written as follows:

(4) $$L=\frac{L_{1}L_{2}}{L_{1}+L_{2}}+L_{0},$$

(5) $$C=\frac{\left(C_{1}+C_{2}\right)C_{0}}{C_{1}+C_{2}+C_{0}}.$$

Based on Equations (4) and (5), the resonant frequency of this coupled structure is $f=1/2\pi \sqrt{LC}$, around which the intense coupling resonances will be excited due to the strong interaction between SHS and HTs. Here, the total length (*l* = *l*_1_ + *l*_2_) of the HTs is set to a fixed value. All structural parameters are set to constants except *l*_1_ and *l*_2_. Based on these conditions, the resonant frequency of this coupled structure is only affected by the coupling position between HTs and SHS. In other words, by simply changing the coupling position, the reflected phase of the sound wave can be controllably tuned. However, this kind of modulation will inevitably result in low space utilization and tunability. In order to overcome these defects, this coupled structure is further deformed and optimised. The HTs are deformed into an annular split cavity so that the whole coupled structure is optimised into a nested structure, as shown in Figure 1g. The inner split cavity can be rotated to any angle *θ* (0° ≤ *θ* ≤ 180°), which will simultaneously change the values of *l*_1_ and *l*_2_. The relationship of *θ*, *l*_1_ and *l*_2_ can be written as follows:

(6) $$l_{1}=\frac{\theta }{2}\left(R+R^{\prime }\right),l_{2}=\frac{2\pi -\theta }{2}\left(R+R^{\prime }\right).$$

Here, *R* and *R′* represent the outer and inner radiuses of the annular cavity, respectively. From Equation (6) we can see that, by only changing the rotation angle of inner split cavity, the resonant frequency and reflected phase of the designed nested structure can be controllably altered.

**Figure 2:**
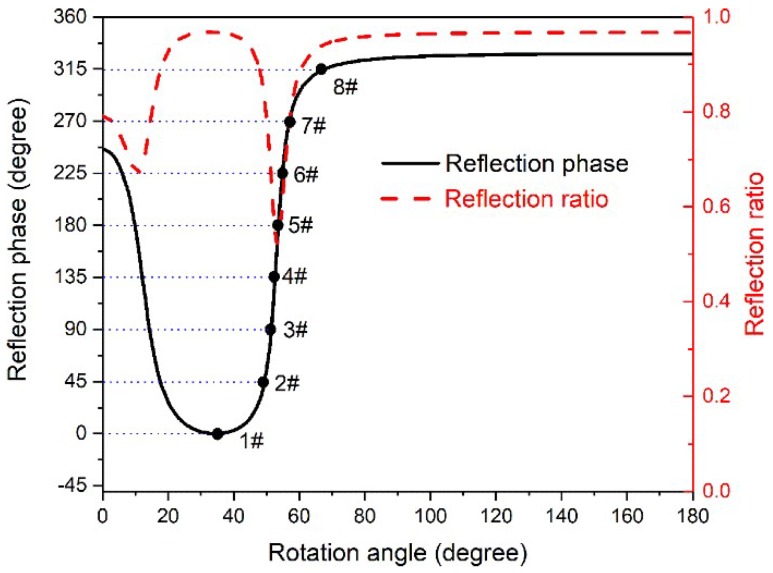
Reflection phase and ratio of the microunit versus rotation angle *θ* of the inner cavity at 2000 Hz. The black dots denote the specific *θ* values of eight microunits to fulfill the desired discrete phase shifts. The *θ* values of these eight microunits are optimised to be 34, 49, 52, 53, 54, 55, 57 and 68°, respectively.

In order to verify the aforementioned theory, the commercial software COMSOL Multiphysics™ 5.2a (lnady Company for Engineering & Agencie, Nasr City, Egypt) was employed to study the reflection behaviour of the proposed nested structure. The outer and inner radiuses of the annular cavity are chosen to be *R* = 20 mm and *R*′ = 14 mm, respectively. The hole widths of both outer and inner cavities are fixed to be *d* = 5 mm. The wall thickness is *h* = 4 mm. Materials used in the simulations are the air as a propagation medium, and the polyethylene plastic as side wall that is hard enough to be acoustic rigid boundary. Considering the viscous loss, the air medium is set to be a viscous fluid with the bulk viscosity of 0.02. The sound speed and mass density of air are *c* = 343 m/s and *ρ* = 1.21 kg/m^3^, respectively. The periodic boundary condition is applied along the direction perpendicular to the incident sound wave to eliminate boundary effects. In addition, the plane wave radiation boundary condition is applied for the incident wave propagating. The operation frequency is 2000 Hz. Figure 2 displays the reflection phase and ratio of this nested structure as a function of rotation angle *θ* of the inner cavity. According to this figure, we verify that the reflection phase can indeed be tuned almost from 0° to 360° by only changing one geometric parameter of this structure. Besides, the overall reflection ratio is acceptable, although a small portion of the reflection ratio is inevitably lowered due to the strong coupling resonance. Under these conditions, the designed nested structure is suitable to act as the microunit for constructing acoustic metasurfaces. Here, eight microunits [23] with different rotation angles are chosen to cover 360° phase shift with an interval of 45° between adjacent microunits, which are labeled with black dots in Figure 2 (i.e., 1#~8#).

### 2.2. Tunable Acoustic Metasurface for High-Q Spectrum Splitting

**Figure 3:**
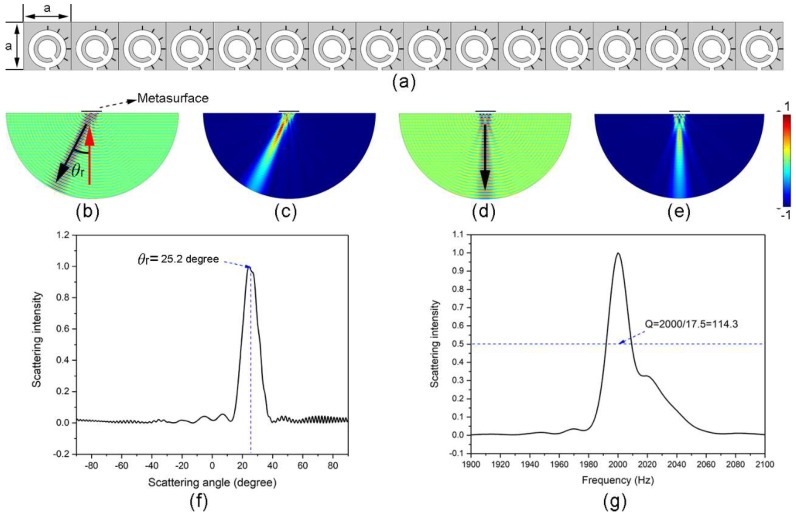
Acoustic metasurface for high-Q spectrum splitting. (**a**) Schematic sketch of the constructed metasurface, which consists of 16 microunits. All the microunits are squares with the same side lengths. The phase gradient interval of adjacent microunits is 45°. The grey areas are acoustic rigid materials, and the white areas are filled with air medium. (**b**) Normalised transient sound pressure field distribution of reflected waves at 2000 Hz. The red and black arrows indicate the propagation directions of incident and reflected waves, separately. (**c**) Normalised squared absolute pressure distribution of reflected waves at 2000 Hz. (**d**) and (**e**) Normalised transient sound pressure and squared absolute pressure field distributions of reflected waves at 1980 Hz for comparison. (**f**) Normalised scattering field intensity of sound wave versus scattering angle at 2000 Hz. (**g**) Normalised scattering field intensity of sound wave of the scattering angle of 25.2° as a function of frequency.

Based on Fermat’s principle, the generalised Snell’s law of reflection can be derived as follows [38]:

(7) $$\sin\theta_{r}-\sin\theta_{i}=\frac{1}{k_{0}}\frac{d\phi }{dx},$$

where *θ*_i_ and *θ*_r_ are the incident and reflection angles, respectively. *k*_0_ = 2π/*λ*_0_ is the wave vector in air. *dϕ*/*dx* is the extra phase gradient on the metasurface. In this work, the incident wave is a plane wave, and the incident angle is kept at a constant *θ*_i_ = 0°. Therefore, the reflection angle of the metasurface is only related to the extra phase gradient, which can be obtained by simply arranging eight microunits in order and at equal intervals. Figure 3a shows the schematic sketch of the metasurface constructed with 16 designed microunits (i.e., 2 periods). To simplify the fabrication of the structure, the external side length of microunits are fixed to a = 50 mm, and adjacent microunits are closely arranged. In this circumstance, the extra phase gradient will be *dϕ*/*dx* = 0.90°/mm. Based on Equation (7), the theoretical reflection angle of this metasurface at the working frequency of 2000 Hz will be 25.4°. In our simulations to study the reflected fields of the metasurface, the incident sound wave is set as background pressure field. The perfectly matched layers are implemented all around the entire solved region to avoid the scattering of the boundaries to sound waves.

Figure 3b,c show the patterns of normalised transient pressure field and squared absolute pressure of reflected sound waves separately at the working frequency. It is obvious that, after the incident wave impinges on the metasurface in the vertical direction, the reflected beam is abnormal. Figure 3f depicts the normalised scattering field intensity of sound wave along the external semicircle boundary versus the scattering angle. The exact reflection angle corresponds to the peak location of the scattered field, which is 25.2°. Good agreement is found between the simulated result and the theoretical value. In order to obtain the working bandwidth of this metasurface, the scattering intensity at *θ*_r_ = 25.2° is studied systematically from 1900 Hz to 2100 Hz, and the result is shown in Figure 3g. The intense coupling resonance effect in the microunit exhibits high sensitivity to the change in frequency. As a result, the half-peak width of the metasurface is as narrow as 17.5 Hz and the Q-factor is as high as 114.3, which is an order of magnitude higher than the previous works [29,31]. In comparison of the abnormal reflection of this metasurface at 2000 Hz, the patterns of normalised transient pressure field and squared absolute pressure of reflected sound waves at non-operating frequency (e.g., 1980 Hz) are shown in Figure 3d,e. The reflected beam is obviously perpendicular to the metasurface, which corresponds to a normal reflection. That is to say, the reflected wave-fronts of this metasurface propagate in two distinct directions within different frequency regions. This also means that the designed metasurface can be regarded as a kind of high-Q spectrum splitter to split sound waves with different frequencies.

**Figure 4:**
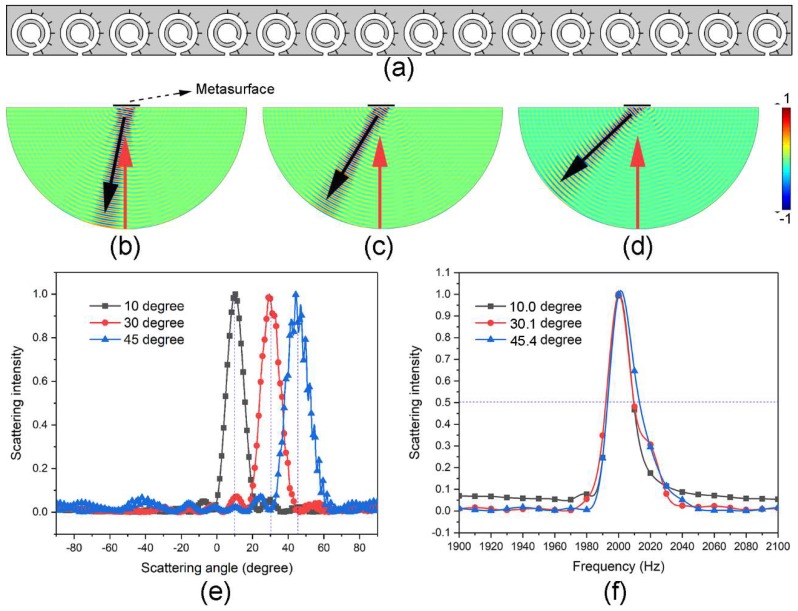
Tunability of the metasurface. (**a**) Schematic sketch of the metasurface for the phase gradient *dϕ*/*dx* = 0.36°/mm. The rotation angles of inner split cavities in 16 microunits are 34, 46.2, 48.8, 50, 51, 51.5, 52, 52.5, 52.9, 53.3, 53.7, 54.2, 54.7, 55.4, 56.3, and 57.6°, respectively. (**b**), (**c**), and (**d**) The normalised transient reflected fields of sound waves of the metasurface configured according to three different phase gradients, respectively, at 2000 Hz. The red and black arrows indicate the propagation directions of incident and reflected waves, separately. (**e**) Scattering field intensities of sound waves of (**b**), (**c**), and (**d**) versus scattering angle. The black, red and blue curves correspond to the target reflected angles of 10, 30, and 45°, respectively. (**f**) Scattering field intensities of sound waves of respective target angle for (**b**), (**c**), and (**d**) as a function of frequency.

We have demonstrated that the reflection phase of microunit can be altered by the rotation of inner split cavity, and the reflection angle of the constructed metasurface is only determined by the phase gradient along the surface. Therefore, the tunability of this metasurface can be realised via mechanically changing the rotation angle of inner split cavity in every microunit according to the requirement. In what follows, another three reflection angles (i.e., 10, 30, and 45°) are achieved to verify the tunability of the metasurface, which is still composed of 16 closely aligned microunits. However, based on Equation (7), the corresponding phase gradients along the surface are 0.36, 1.05, and 1.48°/mm, respectively. In these cases, it cannot meet the demand if we still arrange the microunits with the adjacent phase shifts of 45°. Hence, we need to reselect the microunits with specific reflection phase on the curve in Figure 2 instead of the eight black dots. Take the phase gradient *dϕ*/*dx* = 0.36°/mm for instance, the required reflection phases of 16 microunits are 0, 18, 36, 54, 72, 90, 108, 126, 144, 162, 180, 198, 216, 234, 252, and 270°, respectively. Then, the corresponding rotation angles of inner split cavities in 16 microunits can be easily located on the curve in Figure 2. The schematic sketch of the constructed metasurface is shown in Figure 4a. This design method is also suitable for the cases of *dϕ*/*dx* = 1.05 and 1.48°/mm. Figure 4b–d show the simulated patterns of normalised transient pressure field of reflected sound waves for the selected three phase gradients separately at the working frequency (i.e., 2000 Hz). Apparently, the reflection directions are different. Figure 4e exhibits the comparison result of the normalised scattering field intensity of sound wave along the external semicircle boundary versus the scattering angle for these three phase gradients. The corresponding reflection angles are 10.0, 30.1 and 45.4°, separately, which match well with our preset values. Thus, the tunability of constructed metasurface is verified. Besides, the comparison result of scattering field intensity at respective reflection angles as a function of frequency is shown in Figure 4f. The corresponding Q-factors are 120.9, 112.7, and 100.6, respectively. As for the incident waves out of the operating frequency region, the reflected waves are always perpendicular to the metasurface. This means that the designed metasurface maintains high Q-factors within a wide reflection angle range, and it will be very stable once the metasurface is used for a tunable spectrum splitter. It should be noted that the proposed structure can also be tuned over different working frequencies by adjusting other geometric parameters.

## 3. Conclusions

In summary, by utilizing nested structures integrated by a split cavity and an annular split cavity as building blocks, we achieved a tuneable metasurface in an audible frequency range. Because of the intense coupling resonance effect, the microunit can realise a reflection phase shift covering nearly 2π region, as the inner split cavity rotates around its centre axis from 0° to 180°. As a result, the constructed metasurface operates efficiently in a narrow spectrum and the reflection direction is tuneable based on different phase gradients along the surface. Thus, this metasurface can be used as a tuneable wide-angle spectrum splitter with a high Q-factor. The revealed model in this research may also find applications for the design of tuneable narrow-band acoustic filters, acoustic cloaks and acoustic switching devices.
